# Supplementary material for: Long-term major adverse liver outcomes in 1,260 patients with non-cirrhotic NAFLD
Source: JHEP Rep. 2023 Sep 25;6(2):100915. doi: 10.1016/j.jhepr.2023.100915 (PMC10827505; doi:10.1016/j.jhepr.2023.100915)
Supplement: Multimedia component 1 [file mmc1.pdf]

# Long-term major adverse liver outcomes in 1,260 patients with non-cirrhotic NAFLD

Camilla Akbari, Maja Dodd, Per Stål, Patrik Nasr, Mattias Ekstedt, Stergios Kechagias, Johan Vessby, Fredrik Rorsman, Xiao Zhang, Tongtong Wang, Thomas Jemielita, Gail Fernandes, Samuel S. Engel, Hannes Hagström, Ying Shang

Table of contents

Table S1……………………………………………………………………………2

Table S2……………………………………………………………………………4

Table S3……………………………………………………………………………5

Table S4……………………………………………………………………………6

Table S5……………………………………………………………………………7

**Table S1.** International Classification of Disease (ICD) and Anatomical Therapeutic Chemical Classification System (ATC) codes for liver-related outcome and other liver disease.

| | **ICD10** | **ICD9** | **ICD8** |
|---|---|---|---|
| ***Major adverse liver outcomes*** | | | |
| *Compensated cirrhosis* | | | |
| Liver cirrhosis, unspecified | K74.6 | 571F | 571.9 |
| Esophageal varices (not bleeding) | I85.9, I98.2 | 456B | |
| Gastric varices (not bleeding) | I86.4 | | |
| *Decompensated cirrhosis* | | | |
| Hepatorenal syndrome | K76.7 | 572E | - |
| Ascites | R18.9 without any code for heart failure (I50) or non-HCC cancer (any C, except C22.0) | 789F without any code for heart failure (428) or non-HCC cancer (any 140-209, except 155A) | 785.3 without any code for heart failure (427.0/1, 428.9) or non-HCC cancer (any 140-209, except 155.01) |
| Esophageal varices (bleeding) | I85.0, I98.3 | 456A, 456C | 456.0 |
| Hepatic encephalopathy | K74.6 + ATC code A06AD11/A07AA11 (lactulose/rifaximin) | 572C | |
| *Hepatocellular carcinoma* | C22.0 | 155A | 155.01 |
| *Chronic or unspecific liver failure* | K72.1, K72.9 | 572W | 573 |
| *Liver transplantation* | | | |
| Diagnostic codes | Z94.4 | V42H | - |
| Procedure codes | JJC00, JJC10, JJC20, DJ005, DJ006, JJC30, JJC40 | 5200 | 5200 |
| ***Other liver disease*** | | | |
| Alcohol-related cirrhosis | K70.3 | 571C | 571.00 |
| Viral hepatitis | B16, B17, B18, B19 | 070, 571E | 070, 999.2 |
| Primary biliary cholangitis | K74.3, K74.5 | 571G | - |
| Primary sclerosing cholangitis | (K50 or K51) + K83.0 | (555 or 556) + 576B | 563 + 575.05 |

| | | | |
|---|---|---|---|
| Autoimmune hepatitis | K75.4 | - | - |
| Budd-Chiari syndrome | I82.0, K76.5 | 453A | - |
| Alpha-1 antitrypsin deficiency | E88.0A, E88.0B | 277G | - |
| Wilson’s disease | E83.0B | 275B | 273.30 |
| Hemochromatosis | E83.1 | 275A | 273.20 |

**Table S2.** International Classification of Disease (ICD) codes for comorbidities.

| | ICD10 | ICD9 | ICD8 |
|---|---|---|---|
| Cardiovascular disease | Ischemic heart disease: I20-I25<br>Cerebrovascular disease: I60-I69<br>Peripheral artery disease: I73.9 | Ischemic heart disease: 410-414<br>Cerebrovascular disease: 430-438<br>Peripheral artery disease: 443X | Ischemic heart disease: 410-414<br>Cerebrovascular disease: 430-438<br>Peripheral artery disease: 443.90 |
| Type 2 diabetes | E11 | 250 | 250 |
| Hypertension | I10-I15 | 401-405 | 400-404 |
| Hyperlipidemia | E78 | 272A-E | 272.0/1 |
| Cancer | | | |
| Hepatocellular carcinoma | C220 | 155A | 155.01 |
| Other cancers | C00-C97 (except C220) | 140-208 (except 155A) | 140-209 (except 155.01) |

**Table S3.** Major adverse liver outcomes in patients with NAFLD and the reference population at full follow-up (median 15 years).

*chi-squared test

| | NAFLD, n (%) | Reference population, n (%) | P* |
|---|---|---|---|
| Major adverse liver outcomes | 111 (8.8) | 197 (1.6) | <0.001 |
| Compensated cirrhosis | 58 (4.6) | 51 (0.4) | <0.001 |
| Decompensated cirrhosis | 34 (2.7) | 77 (0.6) | <0.001 |
| Esophageal varices (bleeding) | 8 (0.6) | 15 (0.1) | <0.001 |
| Ascites | 21 (1.7) | 69 (0.6) | <0.001 |
| HRS | 3 (0.2) | 5 (0.0) | <0.001 |
| HE | 19 (1.5) | 1 (0.0) | <0.001 |
| Chronic liver failure | 33 (2.6) | 49 (0.4) | <0.001 |
| HCC | 16 (1.3) | 38 (0.3) | <0.001 |
| Liver transplant | 3 (0.2) | 3 (0.0) | <0.001 |
| Liver-related death | 33 (2.6) | 92 (0.7) | <0.001 |

**Abbreviations:** HCC = hepatocellular cancer; HE = hepatic encephalopathy; HRS = hepatorenal syndrome, NAFLD = nonalcoholic fatty liver disease.

**Table S4**. Anatomical Therapeutic Chemical Classification System (ATC) codes for comorbidities.

| | **ATC** |
|---|---|
| Anti-diabetic medication | A10 |
| Anti-hypertensive medication | C03AA, C07AB, C08C, C09 |
| Statins | C10AA |

**Table S5**. C-index for continuous or age-dependent categorical FIB-4 compared to fibrosis stage estimated by liver biopsy or by biopsy or VCTE when biopsy was not available.

| **C-index for FIB-4 vs. biopsy (n = 904)** | **C statistics *(95% CI)* at 5 years** | **C statistics *(95% CI)* at 10 years** |
|---|---|---|
| **Biopsy** | 0.701 *(0.644-0.733)* | 0.734 *(0.630-0.785)* |
| **FIB-4 continuous** | 0.713 *(0.589-0.760)* | 0.727 *(0.623-0.774)* |
| **FIB-4 categorical** | 0.690 *(0.616-0.722)* | 0.716 (*0.626-0.755)* |
| | | |
| **C-index for FIB-4 vs. VCTE or Biopsy (n = 1,022)** | | |
| **VCTE or biopsy** | 0.724 *(0.634-0.778)* | 0.748 *(0.668-0.802)* |
| **FIB-4 continuous** | 0.695 *(0.619-0.736)* | 0.703 *(0.612-0.754)* |
| **FIB-4 categorical** | 0.710 *(0.624-0.767)* | 0.716 *(0.618-0.764)* |

**Abbreviations:** FIB-4 = fibrosis-4; VCTE = vibration-controlled transient elastography.

FIB-4 category was age-dependent: for age<64: low risk: <1.3, intermediate risk: 1.3-2.67, high risk: >2.67; for age ≥65: low risk: <2.0, intermediate risk: 2.0-2.67, high risk: >2.67
