# Supplementary material for: Long-term major adverse liver outcomes in 1,260 patients with non-cirrhotic NAFLD
Source: JHEP Rep. 2023 Sep 25;6(2):100915. doi: 10.1016/j.jhepr.2023.100915 (PMC10827505; doi:10.1016/j.jhepr.2023.100915)
Supplement: Multimedia component 4 [file mmc4.pdf]

Research article

JHEP Reports

Long-term major adverse liver outcomes in 1,260 patients with non-cirrhotic NAFLD

Camilla Akbari,[1] Maja Dodd,[1] Per Stål,[1,2] Patrik Nasr,[1,3] Mattias Ekstedt,[3] Stergios Kechagias,[3] Johan Vessby,[4] Fredrik Rorsman,[4] Xiao Zhang,[5] Tongtong Wang,[5] Thomas Jemielita,[5] Gail Fernandes,[5] Samuel S. Engel,[5] Hannes Hagström,[1,2,*,#] Ying Shang[1,#]

[1]Department of Medicine, Huddinge, Karolinska Institutet, Stockholm, Sweden; [2]Division of Hepatology, Department of Upper GI, Karolinska University Hospital, Stockholm, Sweden; [3]Division of Internal Medicine, Department of Gastroenterology and Hepatology and Department of Health, Medicine, and Caring Sciences, Linköping University, Linköping, Sweden; [4]Department of Medical Sciences, Gastroenterology Research Group, Uppsala University Hospital, Uppsala, Sweden; [5]Merck & Co., Inc., Rahway, NJ, USA

JHEP Reports **2024.** https://doi.org/10.1016/j.jhepr.2023.100915

Background & Aims: Long-term studies of the prognosis of NAFLD are scarce. Here, we investigated the risk of major adverse liver outcomes (MALO) in a large cohort of patients with NAFLD.
Methods: We conducted a cohort study with data from Swedish university hospitals. Patients (n = 1,260) with NAFLD without cirrhosis were diagnosed through biopsy or radiology, and had fibrosis estimated through vibration-controlled transient elastography, biopsy, or FIB-4 score between 1974 and 2020 and followed up through 2020. Each patient was matched on age, sex, and municipality with up to 10 reference individuals from the general population (n = 12,529). MALO were ascertained from Swedish national registers. The rate of events was estimated by Cox regression.
Results: MALO occurred in 111 (8.8%, incidence rate = 5.9/1,000 person-years) patients with NAFLD and 197 (1.6%, incidence rate = 1.0/1,000 person-years) reference individuals during a median follow up of 13 years. The rate of MALO was higher in patients with NAFLD (hazard ratio = 6.6; 95% CI = 5.2–8.5). The risk of MALO was highly associated with the stage of fibrosis at diagnosis. In the biopsy subcohort (72% of total sample), there was no difference in risk between patients with and without non-alcoholic steatohepatitis. The 20-year cumulative incidences of MALO were 2% for the reference population, 3% for patients with F0, and 35% for F3. Prognostic information from biopsy was comparable to FIB-4 (C-indices around 0.73 *vs.* 0.72 at 10 years).
Conclusions: This study provides updated information on the natural history of NAFLD, showing a high rate of progression to cirrhosis in F3 and a similar prognostic capacity of non-invasive tests to liver biopsy.
Impact and implications: Several implications for clinical care and future research may be noted based on these results. First, the risk estimates for cirrhosis development are important when communicating risk to patients and deciding on clinical monitoring and treatment. Estimates can also be used in updated health-economic evaluations, and for regulatory agencies. Second, our results again highlight the low predictive information obtained from ascertaining NASHstatus by histology and call for more objective means by which to define NASH. Such methods may include artificial intelligence-supported digital pathology. We highlight that NASH is most likely the causal factor for fibrosis progression in NAFLD, but the subjective definition makes the prognostic value of a histological NASH diagnosis of limited value. Third, the finding that prognostic information from biopsy and the very simple Fibrosis-4 score were comparable is important as it may lead to fewer biopsies and further move the field towards non-invasive means by which to define fibrosis and, importantly, use non-invasive tests as outcomes in clinical trials. However, all modalities had modest discriminatory capacity and new risk stratification systems are needed in NAFLD. Repeated measures of non-invasive scores may be a potential solution.
© 2023 Merck Sharp & Dohme LLC., a subsidiary of Merck & Co., Inc., Rahway, NJ, USA and The Author(s). Published by Elsevier B.V on behalf of European Association for the Study of the Liver (EASL) This is an open access article under the CC BY license (http://creativecommons.org/licenses/by/4.0/).

Introduction

NAFLD is a major health problem with risk of progression to cirrhosis, decompensated cirrhosis, and hepatocellular carcinoma (HCC).[1] As only a minority of patients with NAFLD develop such outcomes, estimating this risk is an important part of the patient evaluation. Liver fibrosis has repeatedly been shown to be the most important parameter for estimating the risk of major adverse liver outcomes (MALO) in NAFLD.[2–5] Hence, the detection of fibrosis – in particular those with stage 3-4, termed advanced

Keywords: NAFLD; Non-invasive; NASH; FIB-4; Prediction; Fibrosis stage; Major adverse liver outcomes.
Received 26 August 2023; accepted 6 September 2023; available online 25 September 2023
# Joint senior authors.
* Corresponding author. Address: C1:77, Division of Hepatology, Karolinska University Hospital, 141 86 Stockholm, Sweden. Tel.: +46 8 5858 2305, fax: +46 8 5858 2335.
E-mail address: hannes.hagstrom@ki.se (H. Hagström).

ELSEVIER

EASL The Home of Hepatology

fibrosis – is of high clinical importance, as mentioned in several clinical guidelines.[6–8] The traditional method for evaluating the stage of fibrosis is liver biopsy, which has disadvantages such as invasiveness, a poor intra- and interobserver correlation with sampling variability, and high costs.[9] This highlights the need for alternative non-invasive methods to estimate the stage of fibrosis with similar, or better, prognostic information to that of biopsy. Another debated topic is the importance of histological NASH for prediction of incident cirrhosis. Because NASH is highly collinear with fibrosis stage, it is difficult to tease out the individual contribution of NASH to predicting disease progression.[3,10]

Previous studies that have evaluated the difference in MALO restrictively based on fibrosis stage in patients with NAFLD have usually had limited sample sizes, few hard outcomes, short follow-up time or low granularity. In all, such limitations can lead to unprecise risk estimates that may not be generalisable. Also, the predictive capacity of liver biopsy compared with non-invasive scores such as the commonly used FIB-4 score regarding MALO is a relatively new topic.[7,11] A recent multi-national study suggested that biopsy was superior to FIB-4 scores, but comparable to liver stiffness measurement (LSM) using vibration-controlled transient elastography (VCTE). However, the median follow up was short at around 3 years.[7] Another recent meta-analysis also suggested that non-invasive tests provide similar prognostic information to histologically assessed liver fibrosis.[12] Here, we aimed to investigate the long-term prognosis of a large cohort of patients with NAFLD regarding the risk of MALO across different stages of fibrosis and the presence of NASH. Further, we aimed to compare different methods for staging fibrosis as predictors for such outcomes.

Patients and methods

Study design and study population

We conducted a cohort study (n = 1,333), pooling data from four different cohorts: Fatty Liver In Sweden part 1 (FLIS-1, *n* = 95),[13] Fatty Liver In Sweden part 2 (FLIS-2, *n* = 102), a previously published cohort study (n = 712),[3] and a contemporary cohort through a new data collection by medical chart review (n = 424). FLIS-1 was a multi-centre cross-sectional study at several Swedish university hospitals (Karolinska University Hospital, Linköping University Hospital, Sahlgrenska University Hospital, Uppsala University Hospital, and Skåne University Hospital), originally examining the role of moderate alcohol consumption in NAFLD. All patients underwent liver biopsy as part of the study.[13] FLIS-2 is an ongoing cohort study, including patients with a diagnosis of NAFLD and fibrosis staging either through biopsy or VCTE, and with longitudinal follow-up over 5 years. A description of our previous long-term follow-up study of patients with biopsy-defined NAFLD is available elsewhere.[3] Here, we additionally collected data from patients at Karolinska University Hospital in Stockholm, Linköping University Hospital, and Uppsala University Hospital with a diagnosis of NAFLD (n = 424), who were first identified in each hospital's electronic medical charts using a search for the International Statistical Classification of Diseases 10th Edition (ICD-10) code K76.0.[14] Next, charts from such patients were reviewed by two researchers (CA, MD) to verify the diagnosis and extract data. The diagnosis of NAFLD was made through differing methods including liver biopsy (72%), and radiological measures such as controlled attenuation parameter (CAP), ultrasound, or other radiologic examinations. The fibrosis stage was estimated through biopsy, VCTE if biopsy was missing, or FIB-4 if both biopsy and VCTE were missing.

Exclusion criteria

Patients with presumed NAFLD were excluded if they had liver diseases other than NAFLD at or before baseline during chart review or by register linkage. These included alcohol-related or drug-induced liver injury, autoimmune liver disease, viral hepatitis, cholestasis, or genetic liver disease. Other exclusion criteria included an estimated daily alcohol consumption during chart review of more than 30 g for men or 20 g for women at baseline; binge drinking, defined as reporting a regular consumption of ≥5units of alcohol for men and ≥4 units for women on one and the same occasion; or previous liver decompensation. As we were interested in progression to cirrhosis, patients with baseline cirrhosis, defined as fibrosis stage 4 (FIB-4) on biopsy or VCTE ≥15 kPa, were excluded.[15,16] In patients with NAFLD and the matched reference group, those with register-based diagnoses of cirrhosis, decompensation, HCC, or age <18 years were also excluded. ICD codes used to classify exclusion criteria in registers are presented in Table S1.

Baseline characteristics

Characteristics were collected from patient charts and from register linkages at baseline and at repeated timepoints, where available. This study only used data from baseline. All diagnoses (liver diseases and comorbidities) at the time of inclusion were obtained from patient charts and through ICD codes and Anatomical Therapeutic Chemical Classification System (ATC) codes obtained from registers[17] (definitions in Table S2). Hypertension was defined as a registered diagnosis, a systolic blood pressure ≥140 mmHg or diastolic blood pressure ≥90 mmHg, or the presence of antihypertensive treatment. Type 2 diabetes mellitus was defined as a registered diagnosis in the charts or from the national patient register (NPR),[18] having any anti-diabetic medication prescribed, or having a fasting glucose value of ≥7.0 mmol/L. Hyperlipidaemia was defined as either a registered diagnosis in charts or the NPR, or prescribed treatment with statins or other antilipidaemic treatment, or a fasting total cholesterol value of ≥200 mg/dl. Clinical parameters such as weight and height were measured by healthcare workers within 1 month after inclusion and were used to calculate BMI. Routine biochemical variables within 1 month of liver biopsy were extracted from patient charts. Information about education was collected from registers at Statistics Sweden,[18] and education was defined as <10 years, 10–12 years, or >12 years. Lifestyle factors such as smoking were obtained from patient charts that documented whether the patient was currently a smoker, had been a smoker, or had never smoked. Key medications were obtained from the list of medications reported in the patient charts on the day of inclusion. Several other variables were also collected for every patient at baseline (Table 1).

Histopathological evaluation and modalities to stage liver fibrosis

Liver biopsies were analysed slightly differently depending on which cohort they emanated from. In biopsies from our previously reported cohort study with historical data[3] and the FLIS-1 study,[13] slides were previously reviewed by an expert pathologist (Professor Rolf Hultcrantz, deceased) and one of the authors (HH), after calibrating of the methodology with an internationally recognised expert (Professor Pierre Bedossa). These biopsies

Table 1. Baseline demographic, clinical, and histopathological characteristics of study participants (n = 1,260).

Parameters	Complete data, N	Missingness (%)	Median (IQR)/frequency (%)
Age	1,260	0	52 (39-60)
Sex (male)	1,260	0	748 (59.4)
Sites	1,260	0	
Stockholm			941 (74.7)
Linköping			214 (17.0)
Uppsala			94 (7.5)
Others			11 (0.9)
Education	1,215	3.5	
≤10 years			322 (26.5)
10–12 years			513 (42.2)
>12 years			380 (31.3)
Inclusion period	1,260	0	
1974–1980			39 (3.1)
1981–1990			361 (28.7)
1991–2000			186 (14.8)
2001–2010			283 (22.4)
2011–2020			391 (31.0)
Country of birth	1,260	0	
Sweden			878 (69.7)
Europe outside of Sweden			187 (14.8)
Others			195 (15.4)
Smoking	1,121	11.0	
Never			640 (57.1)
Current			219 (19.5)
Past			262 (23.4)
BMI (kg/m^2)	1,028	18.4	29.1 (26.4–32.5)
Mode of diagnosis	1,260	0	
Biopsy			904 (71.8)
VCTE			118 (9.4)
Clinical			238 (18.9)
Histology	904	28.3	
NASH*	796	36.8	499 (62.7)
Fibrosis stage 0			222 (24.6)
Fibrosis stage 1			372 (41.2)
Fibrosis stage 2			210 (23.3)
Fibrosis stage 3			100 (11.0)
Steatosis grade	750	40.5	
Steatosis grade 1			291 (38.8)
Steatosis grade 2			206 (27.5)
Steatosis grade 3			253 (33.7)
Lobular inflammation	739	41.3	
Lobular inflammation 0			80 (10.8)
Lobular inflammation 1			342 (46.3)
Lobular inflammation 2			251 (34.0)
Lobular inflammation 3			66 (8.9)
Ballooning	727	42.3	
Ballooning 0			240 (33.0)
Ballooning 1			292 (40.1)
Ballooning 2			195 (26.8)
NAS (1–8)	724	42.5	5 (3–6)
Transient elastography	118	90.6	
CAP (dB/m)	42	96.7	328 (287–355)
LSM (kPa)	118	90.6	6.4 (5.1–7.9)
IQR, LSM	95	92.4	1.1 (0.7–1.6)
Success rate (%)	86	93.2	100 (83–100)
Fibrosis stage by VCTE and biopsy	1,022	18.9	
No or mild (F0–1 on biopsy or <10 kPa)			698 (68.3)
Moderate (F2 on biopsy/10–15 kPa)			224 (21.9)
Advanced fibrosis (stage 3 on biopsy)			100 (9.8)
Biochemical variables			
ALT, µkat/L	1,225	2.8	1.15 (0.77–1.77)
AST, µkat/L	1,205	4.4	0.7 (0.51–0.98)
GGT, µkat/L	1,091	13.4	1.04 (0.64–1.90)
TPK, 10^9/L	1,039	17.5	241 (198–286)
ALP, µkat/L	1,018	19.2	1.32 (1.03–1.78)
INR	1,009	19.9	1 (0.9–1)
Albumin, g/L	1,073	14.8	41 (39–44)
Bilirubin, µmol/L	1,153	8.5	10 (8–14)

(*continued on next page*)

Table 1 (*continued*)

Parameters	Complete data, N	Missingness (%)	Median (IQR)/frequency (%)
HbA1c, mmol/mol	175	86.1	40 (36–50)
Fasting glucose, mmol/L	731	42.0	5.6 (5–6.7)
Haemoglobin, g/L	1,062	15.7	148 (139–157)
Ferritin, mg/mol	617	51.0	209 (110–344)
Total cholesterol, mmol/L	703	44.2	5.7 (4.8–6.5)
Triglycerides, mmol/L	654	48.1	1.9 (1.3–2.7)
HDL, mmol/L	237	81.2	1.1 (0.9–1.3)
LDL, mmol/L	202	84.0	2.2 (3.22–3.99)
Potassium, mmol/L	944	25.1	4.1 (3.8–4.3)
CRP, mg/L	615	51.2	4 (1.3–10)
TSH, mU/L	182	85.6	2.1 (1.5–3.1)
Scoring systems			
FIB-4	1,016	19.4	0.97 (0.69–1.51)
Key comorbidities			
Type 2 diabetes	1,260	0	319 (25.3)
Hypertension	1,260	0	833 (66.1)
Hyperlipidaemia	1,260	0	258 (20.5)
CVD	1,260	0	77 (6.1)
Cancer (except HCC)	1,260	0	106 (8.4)
Key medications			
Statins	1,008	20	113 (11.2)
Antihypertensives	1,012	19.7	317 (31.3)
Antidiabetics	1,011	19.8	131 (12.9)

ALT, alanine aminotransferase; ALP, alkaline phosphatase; AST, aspartate aminotransferase; CAP, controlled attenuation parameter; CRP, C-reactive protein; CVD, cardiovascular disease; GGT, gamma-glutamyl transferase; INR, international normalised ratio; LSM, liver stiffness measurement; NAS, NAFLD activity score; TSH, thyroid-stimulating hormone; VCTE, vibration-controlled transient elastography.
* Defined as presence of steatosis, lobular inflammation, and ballooning, or hepatocellular injury, accompanied by fibrosis.

were scored according to the NASH Clinical Research Network, with a 0–3 scale for lobular inflammation and steatosis, and a 0–2 scale for ballooning.[15] The presence of NASH was here defined using the fatty liver inhibition of progression (FLIP) algorithm, requiring at least one point in steatosis, lobular inflammation, and ballooning.[19,20] For FLIS-2 (n = 62 with biopsy) and the contemporary cohort (n = 115 with biopsy), the local pathologists at each site reviewed the slides and the presence of NASH was defined as the 'gestalt' impression from the original pathology report, as the central reading was not available. Fibrosis stage where biopsy was available was scored using the Kleiner or METAVIR classification systems on a 5-point scale (F0–F4), where F4 is defined as cirrhosis.[15,16] To define the stage of fibrosis at baseline, only patients with biopsy or VCTE data were included. Patients were then categorised into three groups: no or mild (stage 0–1 on biopsy or <10 kPa if biopsy was missing), moderate (stage 2 on biopsy or 10–15 kPa if biopsy was missing), and advanced fibrosis (stage 3 on biopsy).

The FIB-4 score was calculated according to the published formula: (age × AST [IU/L])/(platelets [10^9/L] × ALT [IU/L]$^{1/2}$). FIB-4 subgroups were defined as low risk of advanced fibrosis (<1.30), intermediate risk (1.30–2.67), and high risk (>2.67).

Follow up and outcomes

Follow up started at the time of liver biopsy, at VCTE examination if biopsy was not available, or when a clinical diagnosis was first documented in charts when both biopsy and VCTE were unavailable. The primary outcome was the first occurrence of an MALO, defined as an ICD-based diagnosis of cirrhosis, decompensated cirrhosis (bleeding oesophageal varices, ascites, hepatic encephalopathy, or hepatorenal syndrome), hepatocellular carcinoma (HCC), chronic liver failure, or liver-related death[21] (definitions in Table S1). All Swedish citizens have a personal identification number, which is a unique 10-digit code.[22] This was used to identify patients, to access medical charts, and to create a control population and perform register linkages.

Each patient was matched on age, sex, calendar year at baseline, and municipality, with up to 10 reference individuals at baseline, identified from the Swedish Total Population Register. A total of 12,529 matched reference individuals were identified (Fig. 1). All individuals were followed until the occurrence of MALO, or were censored at non-liver death, emigration, or the end of the follow-up period (December 31, 2020). To follow the cohort, we utilised data from three sources: the NPR of Hospital Discharges, the Swedish Cancer Register (SCR), and the Cause of Death Register (CDR). Hospital discharge diagnoses obtained from the NPR have positive predictive values (PPVs) of around 85–95% depending on the diagnosis,[23] and have been specifically validated for diagnoses corresponding to cirrhosis and NAFLD, with PPVs of >90%.[24,25] The SCR contains information on verified solid and non-solid tumours, and the registry is approximately 96% complete.[26] The CDR provides information on the causes of death for all Swedish inhabitants, including those who have died abroad, and it is mandatory for attending physicians to report the underlying cause of death and any related diseases that could have contributed to the individual's death.[27]

Statistical analysis

Participants' characteristics are expressed as medians with IQR or as total numbers with percentages where applicable. Incidence rates of MALO in patients with NAFLD and their respective matched reference population were calculated. Hazard ratios (HRs) and 95% CI were estimated with Cox regression models, using time of follow-up defined in years as the timescale.[28] The analysis was conditioned on the matching variables (age, sex, and municipality), and no other adjustments were made as a result of lack of granular data in the reference individuals. Comparisons were also made in subgroups of patients with

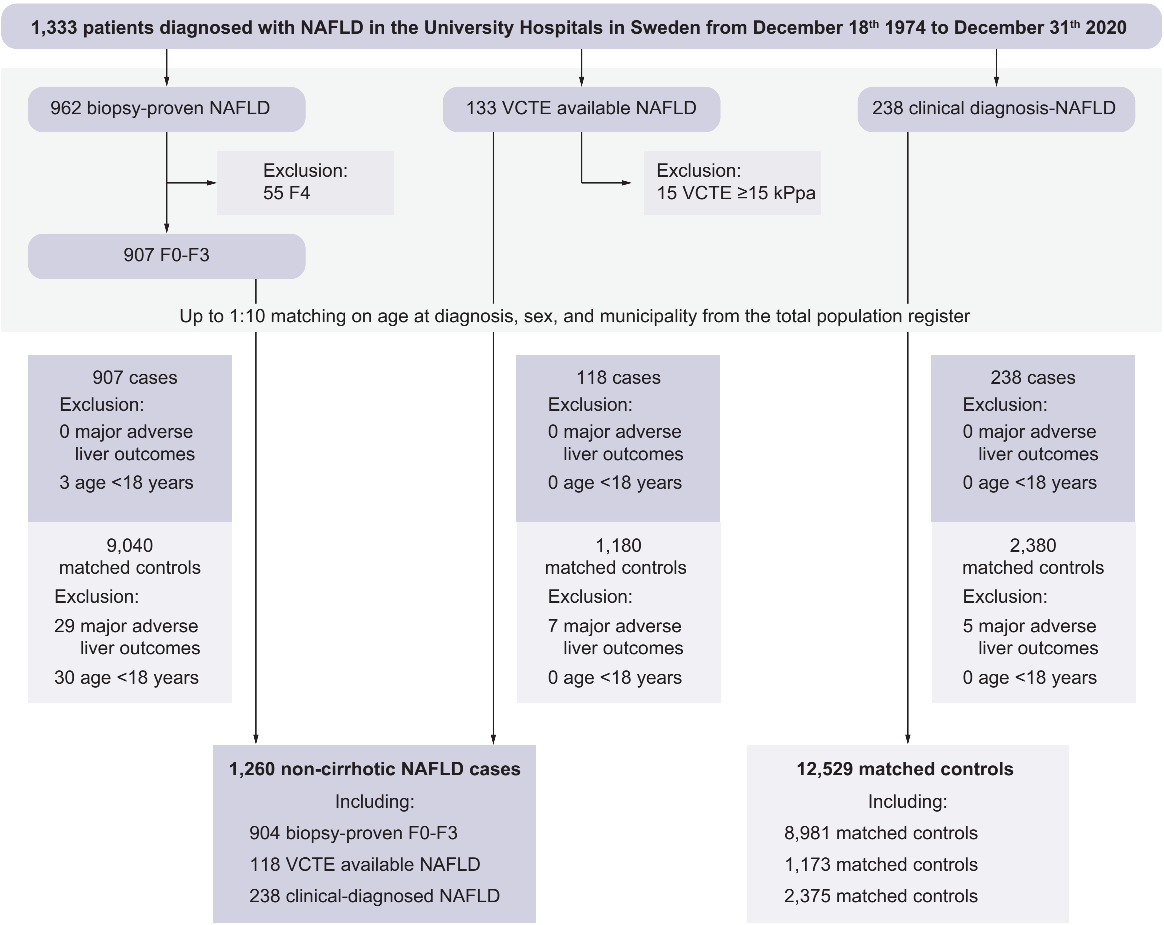

Fig. 1. Flowchart of study participants. VCTE, vibration-controlled transient elastography.

NAFLD, stratified by diagnostic modality (biopsy, VCTE, or clinical), NASH status in those with available data, and fibrosis stages, in all instances comparing patients with NAFLD with their respective matches in the reference population. Cumulative incidences over follow-up time were calculated and plotted by fibrosis severity, accounting for the competing risk of non-liver death by the Aalen-Johansen estimator.[29]

In the biopsy subcohort, we conducted a Cox regression to estimate the HRs of MALO associated with fibrosis stage, using fibrosis stage 0 as the reference group. Similar analyses were also performed among patients with NASH and compared with those without NASH, where possible. Multiplicative interaction was tested between fibrosis stage and NASH status. An 8-level indicator variable was created for fibrosis stage that indicated a potential interaction with NASH (*e.g.* patients with fibrosis stage 2 and NASH), which was then used as an independent variable in another Cox regression model with patients without NASH and fibrosis stage 0 as the reference. A value of p <0.1 indicates a significant interaction. All Cox regression models were adjusted for age and sex, and were additionally adjusted for education, type 2 diabetes, BMI, smoking status, and use of statins. Adjustment factors were decided *a priori* based on clinical knowledge.

Further, we estimated the HRs for MALO using Cox regression models, and computed Harrell's C-index as a measure of model discrimination at 5 and 10 years for each fibrosis staging method. Because not all patients had complete data, we compared the C-index of FIB-4 (continuous and categorical in separate analyses) against those who had biopsy, and against those with either biopsy or VCTE.[30] The 95% CI of the C-index was computed using the bootstrapping method with 500 resamples. We further repeated the analysis using age-related FIB-4 in the sensitivity analysis.

Biochemical variables that exhibited ≤30% missingness were imputed using multiple imputation by chained equations, with five completed datasets generated. Variables with >30% missingness were removed from the analysis altogether. We conducted sensitivity analysis using age-related FIB-4 to assess the impact of missing data on our findings.

Ethical considerations

The study was approved by the Regional Ethical Review Board of Stockholm, with the record number 2018/880-31.

Results

We identified 1,260 patients with NAFLD without cirrhosis and 12,529 matched reference individuals from the general population. Among the 1,260 patients with NAFLD, the median age at baseline was 52 years (IQR: 39–60) and 748 were men (59.4%). A total of 904 patients had a liver biopsy (71.8%), 118 (9.4%) had VCTE but no biopsy, and 238 (18.9%) had neither VCTE nor a biopsy. The median FIB-4 value at baseline was 0.97 (IQR

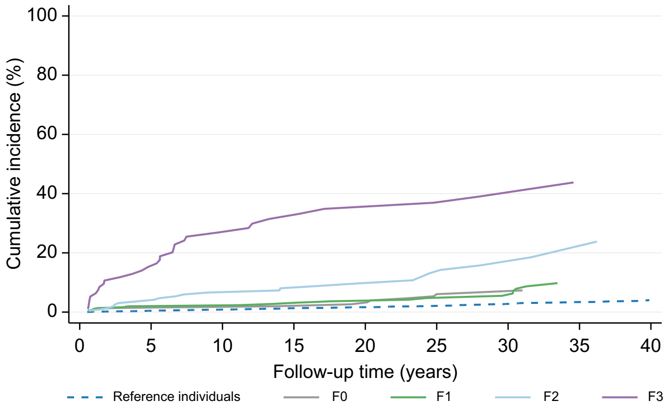

Fig. 2. Cumulative incidence of MALO stratified by fibrosis stage and compared with reference individuals from the general population. MALO, major adverse liver outcomes.

0.69–1.51) and the most common comorbidity was hypertension (n = 833, 66.1%). The median BMI at baseline was 29.1 kg/m^2 (IQR 26.4–32.5) and 15.3% of the population had type 2 diabetes. Within the biopsy group, 499 patients (62.7%) had NASH at baseline. The baseline characteristics are presented in Table 1.

Risk and rate of MALO in patients with NAFLD and the reference population

A total of 111 (8.8%) MALO in the NAFLD group and 197 (1.6%) in the control group ($p \leq 0.001$) occurred during a median follow up of 13.1 years (in total 18,657 and 201,089 person-years of follow up, respectively). The risk of MALO in NAFLD was highly associated with the stage of fibrosis at diagnosis after considering competing risks. The cumulative incidence at 20 years was 3% in patients with F0 and 35% in patients with F3 at baseline (Fig. 2). In contrast, around 2% of reference individuals developed a MALO after 20 years. Cumulative incidence of MALO at 5, 10, and 20 years of follow up are presented in Table 2, stratified by stage of fibrosis at baseline for patients with a biopsy-based diagnosis of NAFLD. The individual outcomes included in the MALO definition for patients with NAFLD and reference individuals are presented in Table S3.

The incidence rate of MALO was 5.9/1,000 person-years (95% CI 4.9–7.2) in the NAFLD population, compared with 1.0/1,000 person-years (95% CI 0.9–1.1) in the reference population. This translated to a HR of 6.6 (95% CI = 5.2–8.5). In the biopsy subcohort, patients with NASH tended to have a numerically higher rate of MALO compared with the reference population (HR 6.7; 95% CI 4.6–9.5) in comparison with those without NASH (HR 4.2; 95% CI 2.5–7.2) (Table 3). However, within the NAFLD population, there was little difference in the rate of MALO when stratified by fibrosis stage and comparing patients with and without NASH (Table 4). We found no evidence of effect modification on the risk of MALO based on presence of NASH in patients with stages 0–2. However, for patients with fibrosis stage 3, the rate of MALO was higher in patients without NASH than in those with NASH, with some evidence of statistical interaction (p = 0.018). However, this was based on only 10 patients in the subgroup of patients with fibrosis stage 3 and no NASH.

Predictivity capacity of FIB-4 against biopsy or VCTE for MALO

Within the NAFLD group, higher fibrosis stages were associated with an increased rate of MALO across all three fibrosis staging modalities. The adjusted hazard ratio (aHR) for F2, as determined by biopsy, was 2.9 (95% CI 1.5–5.5), and for F3 the aHR was 8.9 (95% CI 4.6–17.4), both compared with the reference group with F0–1. In cases of moderate fibrosis defined by biopsy or VCTE (stage 2 on biopsy or 10–15 kPa on VCTE) the aHR was 2.8 (95% CI 1.5–5.2), and for advanced fibrosis (stage 3 on biopsy or ≥15 kPa on VCTE) the aHR was 7.9 (95% CI 4.2–14.8), both compared with the reference group with no or mild fibrosis (stage 0–1 on biopsy or <10 kPa on VCTE). Furthermore, we found a similar association between fibrosis estimated with FIB-4 and incident MALO, with an aHR of 4.7 (95% CI = 2.4–9.1) for those at high risk and an aHR of 2.0 (95% CI = 1.1–3.6) for intermediate risk, compared with patients defined as low risk according to FIB-4 (Table 5).

When examining the discriminative capacity of the three modalities (biopsy; biopsy or VCTE; and FIB-4) for estimating fibrosis stage and incident MALO restricted to the population where all data were available, we found that the C-index was similar for these modalities at 5 and 10 years of follow-up. Fibrosis estimated by biopsy or VCTE demonstrated the highest C-index statistics among the three modalities, followed by biopsy alone and continuous FIB-4 at both 5 and 10 years. However, the categorical FIB-4 group had a similar C-index statistics at 5 years (0.701) compared with the biopsy group (0.701). This trend shifted after 10 years, when the C-index statistics for the categorical FIB-4 group were somewhat lower at 0.719 compared with the biopsy C-index statistics at 0.734 (Table 6). Furthermore, we used age-related cut-off of FIB-4 and found that the C-index was not superior to the FIB-4 without age adjustment (Table S5).

Discussion

Several observations can be made from this large cohort study. First, we confirm that fibrosis stage in NAFLD is predictive of progression to cirrhosis or complications thereof. Because of the large sample size, our estimates may be more accurate than those from previous studies on the topic.[3,31] In fact, we found

Table 2. Cumulative incidence of major adverse liver outcomes stratified by fibrosis stage and compared with reference individuals from the general population.

	1 year		5 years		10 years		20 years	
	No. of events	Cum. inc. (%)	No. of events	Cum. inc. (%)	No. of events	Cum. inc. (%)	No. of events	Cum. inc. (%)
Reference individuals	17	0.13	54	0.44	93	0.85	148	1.66
F0	2	0.91	3	1.38	3	1.38	5	2.63
F1	4	1.08	7	1.96	7	1.96	11	3.66
F2	1	0.11	7	3.59	12	6.59	16	9.67
F3	5	5.26	14	15.2	23	26.9	28	34.9

Cum. inc., cumulative incidence.

Table 3. Associations with MALO in patients with NAFLD and the reference population, for the full population and across subgroups.

	Patients with NAFLD, n	Reference population, n	Events in patients with NAFLD, n	Events in reference population, n	IR/1,000 PYs, patients with NAFLD (95% CI)	IR/1,000 PYs, reference population (95% CI)	Crude HR* (95% CI)
NAFLD, all	1,260	12,529	111	197	5.9 (4.9–7.2)	1.0 (0.9–1.1)	6.6 (5.2-8.5)
NAFLD, biopsy available	904	8,981	84	168	5.4 (4.4–6.7)	1.0 (0.9–1.2)	5.8 (4.3-7.7)
NAFLD, VCTE available	118	1,173	6	6	10.7 (4.8–23.8)	1.1 (0.5–2.4)	9.4 (3.0-29.1)
NAFLD, no biopsy or VCTE	238	2,375	21	23	8.0 (5.2–12.3)	0.6 (0.5–1.6)	12.9 (6.6-24.9)
**In the biopsy subcohort**							
NASH	499	4,952	53	102	6.4 (4.9–8.4)	1.1 (0.9–1.4)	6.7 (4.6-9.5)
No NASH	297	2,962	22	52	4.0 (2.7–6.1)	0.9 (0.7–1.2)	4.2 (2.5-7.2)
F0	222	2,218	11	39	2.4 (1.3–4.3)	0.8 (0.6–1.1)	2.9(1.5-5.8)
F1	372	3,665	19	71	2.9 (1.9–4.6)	1.0 (0.8–1.3)	2.9 (1.6-4.9)
F2	210	2,105	23	35	7.1 (4.7–10.6)	1.0 (0.7–1.4)	8.0 (4.5-14.3)
F3	100	983	31	23	28.2(19.8–40.0)	1.5 (1.0–2.3)	18.4 (9.9-34.2)

HR, hazard ratio; IR, incidence rate; MALO, major adverse liver outcomes; PYs, person-years.
* Matched on municipality, age, and sex.

Table 4. Associations between fibrosis stage and NASH, and MALO, restricted to patients with NAFLD and biopsy data with NASH status available.

	Patients with NAFLD, n	Events in patients with NAFLD, n	IR/1,000 PYs, patients with NAFLD (95% CI)	Crude HR* (95% CI)	Adjusted HR† (95% CI)
**In patients with NASH status available (n = 796)**					
NASH	499	53	6.4 (4.9–8.4)	1.6 (1.0–2.7)	1.8 (0.9–3.3)
No NASH	297	22	4.0 (2.7–6.1)	1.0 (Ref.)	1.0 (Ref.)
**All patients with fibrosis stage available (n = 904)**					
F0	222	11	2.4 (1.3–4.3)	1.0 (Ref.)	1.0 (Ref.)
F1	372	19	2.9 (1.9–4.6)	1.3 (0.6–2.6)	2.0 (0.7–6.2)
F2	210	23	7.1 (4.7–10.6)	3.2 (1.6–6.6)	4.8 (1.6–14.6)
F3	100	31	28.2 (19.8–40.0)	12.6 (6.3–25.3)	15.1 (5.0–46.1)
**In patients with both fibrosis stage and NASH status available (n = 797)**					
No NASH (n = 297)	297	22			
F0	124	6	2.4 (1.1–5.3)	1.0 (Ref.)	1.0 (Ref.)
F1	126	6	2.6 (1.2–5.8)	1.1 (0.4–3.4)	2.5 (0.5–12.8)
F2	37	4	7.3 (2.7–19.5)	3.2 (0.9–11.3)	5.6 (0.9–34.2)
F3	10	6	118.8 (53.4–264.5)	51.5 (15.9–166.3)	100 (17.8–564)
NASH (n = 500)	499	53			
F0	58	4	3.3 (1.2–8.8)	1.4 (0.4–4.9)	2.7 (0.4–19.2)
F1	214	12	3.2 (1.8–5.7)	1.4 (0.5–3.8)	3.0 (0.6–14.2)
F2	151	16	6.6 (4.1–10.8)	3.2 (1.2–8.2)	7.0 (1.5–31.8)
F3	76	21	23.2 (15.1–53.6)	10.8 (4.2–27.2)	19.6 (4.3–89.1)

HR, hazard ratio; IR, incidence rate; PYs, person-years.
* HR adjusted for age and sex.
† HR adjusted for age, sex, education, diabetes, BMI, smoking, and statins.

Table 5. Association between fibrosis estimated by liver biopsy; liver biopsy or VCTE; and FIB-4 in patients with NAFLD and incident major adverse liver outcomes.

Within NAFLD	Patients with NAFLD, n	Events in patients with NAFLD, n	Crude HR† (95% CI)	HR‡ (95% CI)
By biopsy				
F0–1	594	30	1.0 (Ref.)	1.0 (Ref.)
F2	210	23	2.6 (1.5–4.5)	2.9 (1.5–5.5)
F3	100	31	10.9 (6.6–18.4)	8.9 (4.6–17.4)
By biopsy or VCTE				
No or mild fibrosis	698	34	1.0 (Ref.)	1.0 (Ref.)
Moderate fibrosis	224	25	2.5 (1.5–4.2)	2.8 (1.5–5.2)
Advanced fibrosis	100	31	8.4 (5.1–13.9)	7.9 (4.2–14.8)
FIB-4 (continuous)	1,260	111	1.0 (0.9–1.0)	1.1 (1.0–1.2)
FIB-4 (categorical)				
Low (<1.30)	854	51	1.0 (Ref.)	1.0 (Ref.)
Intermediate (1.30-2.67)	324	38	1.8 (1.1–2.9)	2.0 (1.1–3.6)
High (>2.67)	82	22	4.3 (2.4–7.8)	4.7 (2.4–9.1)

FIB-4, fibrosis-4; HR, hazard ratio; VCTE, vibration-controlled transient elastography.
† HR adjusted for age, sex.
‡ HR additionally adjusted for education, diabetes, BMI, smoking, and statins. *p = 0.09.

Table 6. C-index for FIB-4 compared to fibrosis stage estimated by liver biopsy or by biopsy or VCTE when biopsy was not available.

	C statistics (95% CI) at 5 years	C statistics (95% CI) at 10 years
**C-index for FIB-4 *vs.* biopsy (n = 904)**		
Biopsy	0.701 (0.644–0.733)	0.734 (0.630–0.785)
FIB-4 continuous	0.713 (0.589–0.760)	0.727 (0.623–0.774)
FIB-4 categorical	0.701 (0.592–0.752)	0.719 (0.596–0.779)
**C-index for FIB-4 *vs.* VCTE or biopsy (n = 1,022)**		
VCTE or biopsy	0.724 (0.634–0.778)	0.748 (0.668–0.802)
FIB-4 continuous	0.695 (0.619–0.736)	0.703 (0.612–0.754)
FIB-4 categorical	0.729 (0.623–0.788)	0.734 (0.621–0.787)

FIB-4, fibrosis-4; VCTE, vibration-controlled transient elastography.

that more than one-third of patients with fibrosis stage 3 developed cirrhosis within 20 years of follow up. Few previous studies have had this unprecedented duration of follow up. Second, we again confirm a high correlation between the presence of NASH and higher stages of fibrosis. However, within the same stage of fibrosis, we did not find a meaningful difference in risk between patients with and without histological NASH. This may be because of the known issues of uncertainty surrounding the identification of NASH,[32] but could also be because of the low number of patients in the subgroup. Finally, we show that the predictive capacity of different modalities for estimating the stage of fibrosis are comparable when trying to estimate future risk of cirrhosis in NAFLD. This information is important, as it would allow for a transition to the use of non-invasive methods of fibrosis staging when determining risk of future cirrhosis in NAFLD.

Comparison with previous studies

These results are in alignment with previous studies from us[3] and others.[2,4] We also confirm previous findings[2–4,31,33] that presence of NASH, as currently defined by pathologists, does not add much to the prognostic information about the risk of cirrhosis on top of knowledge of the fibrosis stage. This may be because of the subjectivity involved in defining hepatocellular ballooning in particular.[32] In contrast to a recent study with a similar methodology,[7] we found somewhat lower predictive capacity of both biopsy and FIB-4 in terms of lower C-index statistics, and found that estimates from these modalities did not differ significantly. The C-index statistics for FIB-4 and biopsy were 0.71 and 0.70 at 5 years, compared to C-index statistics of 0.93 for biopsy and 0.78 for FIB-4 in the study by Boursier *et al.*[7] This may likely be explained by a considerably longer follow-up in this study, a larger sample size, and more events. Hence, our estimates may be more accurate and generalisable for long-term prediction of MALO.

Strengths and limitations

The main strength of this study is the large size of the cohort – one of the largest cohorts with biopsy-proven NAFLD patients with granular data – and the long follow-up time, allowing enough MALO to be captured to give a meaningful statistical analysis. The linkage to national registers[23] minimises loss to follow up and allows for accurate ascertainment of validated MALO.[25] We could also compare risk estimates to those of matched reference individuals, allowing for a higher level of contextuality. We utilised more advanced statistical techniques than those usually performed in the field, such as multiple imputation to better account for missing data.[34]

Limitations include the fact that NASH status was defined differently depending on which cohort patients were identified from. However, most patients had a biopsy reviewed by an expert pathologist. Because we combined data from four cohorts that were conducted at multiple centres and initiated over different periods, we cannot eliminate the possibility of cohort bias in our study. Liver biopsy was more commonly used in earlier parts of the study period for diagnosing and staging NAFLD, whereas VCTE has only become available in recent years. As a result, it is possible that patient populations may differ between these different modalities, potentially leading to differences in the distribution of patient characteristics over the study period.[35] Secondly, there is always a possibility of misclassification bias in register-based studies. However, the MALO used in this study have been previously validated and found to have high PPVs.[25] Selection bias is always likely in studies with biopsy-diagnosed NAFLD, partly because most patients come from secondary or tertiary care hospitals. Biopsy is not performed in most patients with NAFLD. However, estimates should be comparable to those for other patients in secondary or tertiary levels of care,[7] and the prevalence of advanced fibrosis in this cohort was smaller than in other contemporary cohorts, suggesting a lower risk of selection bias. Thirdly, we did not consider time-dependent variables such as incident T2D and alcohol use in the models. However, such information would not be available at the time of an examination and therefore do not impact the predicted risk from baseline, that is, the diagnosis date of NAFLD. Finally, as the study population consists of patients mainly born in Sweden, the findings may not be generalisable to settings outside Sweden with different risk profiles and ethnicities. As only 5% of the study participants had a BMI <25 kg/m^2, the findings might not apply to this group because of potentially different underlying pathology in patients with lean NAFLD.

Conclusions

In this large cohort study, including 1,260 patients with non-cirrhotic NAFLD, we found that more than one-third of patients with fibrosis stage 3 develop cirrhosis within 20 years. Further, we confirm the role of fibrosis staging in determining prognosis and again show that histological NASH provides little prognostic information. Finally, we found that the prognostic information from histologically defined fibrosis was comparable to the FIB-4 score. However, both modalities had moderate discriminations, and new prediction models for NAFLD are needed.

Abbreviations

aHR, adjusted hazard ratio; CAP, controlled attenuation parameter; CDR, Cause of Death Register; FIB-4, fibrosis-4; FLIP, fatty liver inhibition of progression; FLIS-1, Fatty Liver In Sweden part 1; FLIS-2, Fatty Liver In Sweden part 2; HCC, hepatocellular carcinoma; HR, hazard ratio; ICD, International Classification of Diseases; LSM, liver stiffness measurement; MALO, major adverse liver outcomes; NPR, National Patient Register; PPV, positive predictive values; PYs, person-years; SCR, Swedish Cancer Register; VCTE, vibration-controlled transient elastography.

Financial support

This study was partly funded by a research grant from MSD to Karolinska Institutet (HH). HH was further supported by grants from The Swedish Research Council, The Swedish Cancer Foundation, Region Stockholm and The Åke Wiberg Foundation.

Conflicts of interest

HH's institutions have received research funding from AstraZeneca, EchoSens, Gilead, Intercept, MSD, Novo Nordisk and Pfizer. He has served as a consultant for AstraZeneca and Novo Nordisk, and has been or is part of hepatic events adjudication committees for Boehringer Ingelheim, KOWA and GW Pharma. XZ, TJ, GF, and SSE are employees of Merck Sharp & Dohme LLC, a subsidiary of Merck & Co., Inc., Rahway, NJ, USA, and may own stock and/or hold stock options in Merck & Co., Inc., Rahway, NJ, USA. TW was an employee of Merck Sharp & Dohme LLC, a subsidiary of Merck & Co., Inc., Rahway, NJ, USA, while conducting this work, and may own stock and/or hold stock options in Merck & Co., Inc., Rahway, NJ, USA. She is currently an employee of Johnson & Johnson, and may own stock and/or hold stock options in the company.

Please refer to the accompanying ICMJE disclosure forms for further details.

Authors' contributions

Study concept and design: TW, TJ, HH, YS. Acquisition of data: CA, PN, JV, MD, SK, FR, PS, ME, HH. Statistical analysis: YS. Analysis and interpretation of data: All authors. Drafting of manuscript: CA, HH, YS. Critical revision: All. Guarantor of article: HH. Approved the final version of the article, including the authorship list: All authors.

Data availability statement

Data are subject to personal information protection regulations and are not publicly available. Sharing of anonymised data will be considered on a case-by-case basis on request.

Supplementary data

Supplementary data to this article can be found online at https://doi.org/10.1016/j.jhepr.2023.100915.

References

[1] Younossi ZM, Koenig AB, Abdelatif D, Fazel Y, Henry L. Global epidemiology of nonalcoholic fatty liver disease – meta-analytic assessment of prevalence, incidence, and outcomes. Hepatology 2016;64:73–84.
[2] Angulo P, Kleiner D, Dam-Lersen S, Adams LA, Bjornsson ES, Charatcharoenwitthaya P, et al. Liver fibrosis, but no other histologic features, is associated with long-term outcomes of patients with nonalcoholic fatty liver disease. Gastroenterology 2015;149:389–397. e10.
[3] Hagström H, Nasr P, Ekstedt M, Hammar U, Stål P, Hultcrantz R, et al. Fibrosis stage but not NASH predicts mortality and time to development of severe liver disease in biopsy-proven NAFLD. J Hepatol 2017;67:1265–1273.
[4] Taylor RS, Taylor J, Bayliss S, Hagström H, Nasr P, Schattenberg JM, et al. Association between fibrosis stage and outcomes of patients with nonalcoholic fatty liver disease: a systematic review and meta-analysis. Gastroenterology 2020;158:1611–1625.e12.
[5] Loomba R, Wong R, Fraysee J, Shreay S, Li S, Harrison S, et al. Nonalcoholic fatty liver disease progression rates to cirrhosis and progression of cirrhosis to decompensation and mortality: a real world analysis of Medicare data. Aliment Pharmacol Therapeut 2020;51:1149–1159.
[6] Younossi Z, Anstee Q, Marietti M, Hardy T, Henry L, Eslam M, et al. Global burden of NAFLD and NASH: trends, predictions, risk factors and prevention. Nat Rev Gastroenterol Hepatol 2018;15:11–20.
[7] Boursier J, Hagström H, Ekstedt M, Moreau C, Bonacci M, Cure S, et al. Non-invasive tests accurately stratify patients with NAFLD based on their risk of liver-related events. J Hepatol 2022;76:1013–1020.
[8] Powell EE, Wong VWS, Rinella M. Non-alcoholic fatty liver disease. Lancet 2021;397:2212–2224.
[9] Sumida Y, Nakajima A, Itoh Y. Limitations of liver biopsy and non-invasive diagnostic tests for the diagnosis of nonalcoholic fatty liver disease/nonalcoholic steatohepatitis. World J Gastroenterol 2014;20:475–485.
[10] Wang Y, Hou JL. Current strategies for quantitating fibrosis in liver biopsy. Chin Med J (Engl) 2015;128:252–258.
[11] Barbosa JV, Milligan S, Frick A, Broestl J, Younossi Z, Afdhal NH, et al. Fibrosis-4 index as an independent predictor of mortality and liver-related outcomes in NAFLD. Hepatol Commun 2022;6:765–779.
[12] Mózes F, Lee J, Vali Y, Alzoubi O, Staufer K, Trauner M, et al. Performance of non-invasive tests and histology for the prediction of clinical outcomes in patients with non-alcoholic fatty liver disease: an individual participant data meta-analysis. Lancet Gastroenterol Hepatol 2023;8:704–713.
[13] Hagström H, Nasr P, Ekstedt M, Kechagias S, Önnerhag K, Nilsson E, et al. Low to moderate lifetime alcohol consumption is associated with less advanced stages of fibrosis in non-alcoholic fatty liver disease. Scand J Gastroenterol 2017;52:159–165.
[14] O'Malley KJ, Cook KF, Price MD, Wildes KR, Hurdle JF, Ashton CM, et al. Measuring diagnoses: ICD code accuracy. Health Serv Res 2005;40:1620–1639.
[15] Kleiner DE, Brunt EM, Van Natta M, Behling C, Contos MJ, Cummings OW, et al. Design and validation of a histological scoring system for nonalcoholic fatty liver disease. Hepatology 2005:411313–411321.
[16] Kleiner DE. Histopathology, grading and staging of nonalcoholic fatty liver disease. Minerva Gastroenterol Dietol 2018;64:28–38.
[17] ATC/DDD Toolkit. https://www.who.int/tools/atc-ddd-toolkit/atc-classification Accessed 7 November 2023.
[18] Statistics Sweden. https://www.scb.se/en/ Accessed 7 November 2023.
[19] Bedossa P, FLIP Pathology Consortium. Utility and appropriateness of the fatty liver inhibition of progression (FLIP) algorithm and steatosis, activity, and fibrosis (SAF) score in the evaluation of biopsies of nonalcoholic fatty liver disease. Hepatology 2014;60:565–575.
[20] Bedossa P, Poitou C, Veyrie N, Bouillot JL, Basdevant A, Paradis V, et al. Histopathological algorithm and scoring system for evaluation of liver lesions in morbidly obese patients. Hepatology 2012;56:1751–1759.
[21] Hagström H, Adams L, Allen A, et al. Administrative coding in electronic health care record-based research of NAFLD: an expert panel consensus statement. Hepatology 2021;74:474–482.
[22] Ludvigsson JF, Otterblad-Olausson P, Pettersson BU, et al. The Swedish personal identity number: possibilities and pitfalls in healthcare and medical research. Eur J Epidemiol 2009;24:659–667.
[23] Ludvigsson JF, Andersson E, Ekbom A, Feychting M, Kim JL, Reuterwall C, et al. External review and validation of the Swedish national inpatient register. BMC Public Health 2011;11:450.
[24] Åström H, Wester A, Hagström H. Administrative coding for non-alcoholic fatty liver disease is accurate in Swedish patients. Scand J Gastroenterol 2023;58:931–936.
[25] Bengtsson B, Askling J, Ludvigsson JF, et al. Validity of administrative codes associated with cirrhosis in Sweden. Scand J Gastroenterol 2020;55:1205–1210.
[26] Barlow L, Westergren K, Holmberg L, Talbäck M. The completeness of the Swedish Cancer Register – a sample survey for year 1998. Acta Oncol 2009;48:27–33.
[27] Brooke HL, Talbäck M, Hörnblad J, Johansson LA, Ludvigsson JF, Druid H, et al. The Swedish cause of death register. Eur J Epidemiol 2017;32:765–773.
[28] Stel VS, Dekker FW, Tripepi G, Zoccali C, Jager KJ, et al. Survival analysis II: Cox regression. Nephron Clin Pract 2011;119:c255–c260.
[29] Putter H, Spitoni C. Non-parametric estimation of transition probabilities in non-Markov multi-state models: the landmark Aalen-Johansen estimator. Stat Methods Med Res 2018;27:2081–2092.
[30] Antolini L, Boracchi P, Biganzoli E. A time-dependent discrimination index for survival data. Stat Med 2005;24:3927–3944.
[31] Ekstedt M, Hagström H, Nasr P, Fredrikson M, Stål P, Kerchagias S, et al. Fibrosis stage is the strongest predictor for disease-specific mortality in NAFLD after up to 33 years of follow-up. Hepatology 2015;61:1547–1554.
[32] Brunt EM, Clouston A, Goodman Z, Guy C, Kleiner DE, Lackner C, et al. Complexity of ballooned hepatocyte feature recognition: defining a training atlas for artificial intelligence-based imaging in NAFLD. J Hepatol 2022;76:1030–1041.
[33] Söderberg C, Stål P, Askling J, Glaumann H, Lidberg G, Marmur J, et al. Decreased survival of subjects with elevated liver function tests during a 28-year follow-up. Hepatology 2010;51:595–602.
[34] Azur MJ, Stuart EA, Frangakis C, Leaf PJ. Multiple imputation by chained equations: what is it and how does it work? Int J Methods Psychiatr Res 2011;20:40–49.
[35] Tapper EB, Loomba R. Noninvasive imaging biomarker assessment of liver fibrosis by elastography in NAFLD. Nat Rev Gastroenterol Hepatol 2018;15:274–282.

Journal of Hepatology, Volume *6*

Supplemental information

Long-term major adverse liver outcomes in 1,260 patients with non-cirrhotic NAFLD

Camilla Akbari, Maja Dodd, Per Stål, Patrik Nasr, Mattias Ekstedt, Stergios Kechagias, Johan Vessby, Fredrik Rorsman, Xiao Zhang, Tongtong Wang, Thomas Jemielita, Gail Fernandes, Samuel S. Engel, Hannes Hagström, and Ying Shang

Long-term major adverse liver outcomes in 1,260 patients with non-cirrhotic NAFLD

Camilla Akbari, Maja Dodd, Per Stål, Patrik Nasr, Mattias Ekstedt, Stergios Kechagias, Johan Vessby, Fredrik Rorsman, Xiao Zhang, Tongtong Wang, Thomas Jemielita, Gail Fernandes, Samuel S. Engel, Hannes Hagström, Ying Shang

Table of contents

Table S1……………………………………………………………………………………2

Table S2……………………………………………………………………………………4

Table S3……………………………………………………………………………………5

Table S4……………………………………………………………………………………6

Table S5……………………………………………………………………………………7

Table S1. International Classification of Disease (ICD) and Anatomical Therapeutic Chemical Classification System (ATC) codes for liver-related outcome and other liver disease.

	**ICD10**	**ICD9**	**ICD8**
Major adverse liver outcomes			
Compensated cirrhosis			
Liver cirrhosis, unspecified	K74.6	571F	571.9
Esophageal varices (not bleeding)	I85.9, I98.2	456B	
Gastric varices (not bleeding)	I86.4		
Decompensated cirrhosis			
Hepatorenal syndrome	K76.7	572E	-
Ascites	R18.9 without any code for heart failure (I50) or non-HCC cancer (any C, except C22.0)	789F without any code for heart failure (428) or non-HCC cancer (any 140-209, except 155A)	785.3 without any code for heart failure (427.0/1, 428.9) or non-HCC cancer (any 140-209, except 155.01)
Esophageal varices (bleeding)	I85.0, I98.3	456A, 456C	456.0
Hepatic encephalopathy	K74.6 + ATC code A06AD11/A07AA11 (lactulose/rifaximin)	572C	
Hepatocellular carcinoma	C22.0	155A	155.01
Chronic or unspecific liver failure	K72.1, K72.9	572W	573
Liver transplantation			
Diagnostic codes	Z94.4	V42H	-
Procedure codes	JJC00, JJC10, JJC20, DJ005, DJ006, JJC30, JJC40	5200	5200
Other liver disease			
Alcohol-related cirrhosis	K70.3	571C	571.00
Viral hepatitis	B16, B17, B18, B19	070, 571E	070, 999.2
Primary biliary cholangitis	K74.3, K74.5	571G	-
Primary sclerosing cholangitis	(K50 or K51) + K83.0	(555 or 556) + 576B	563 + 575.05

Autoimmune hepatitis	K75.4	-	-
Budd-Chiari syndrome	I82.0, K76.5	453A	-
Alpha-1 antitrypsin deficiency	E88.0A, E88.0B	277G	-
Wilson’s disease	E83.0B	275B	273.30
Hemochromatosis	E83.1	275A	273.20

Table S2. International Classification of Disease (ICD) codes for comorbidities.

	ICD10	ICD9	ICD8
Cardiovascular disease	Ischemic heart disease: I20-I25<br>Cerebrovascular disease: I60-I69<br>Peripheral artery disease: I73.9	Ischemic heart disease: 410-414<br>Cerebrovascular disease: 430-438<br>Peripheral artery disease: 443X	Ischemic heart disease: 410-414<br>Cerebrovascular disease: 430-438<br>Peripheral artery disease: 443.90
Type 2 diabetes	E11	250	250
Hypertension	I10-I15	401-405	400-404
Hyperlipidemia	E78	272A-E	272.0/1
Cancer			
Hepatocellular carcinoma	C220	155A	155.01
Other cancers	C00-C97 (except C220)	140-208 (except 155A)	140-209 (except 155.01)

Table S3. Major adverse liver outcomes in patients with NAFLD and the reference population at full follow-up (median 15 years).

*chi-squared test

	NAFLD, n (%)	Reference population, n (%)	P*
Major adverse liver outcomes	111 (8.8)	197 (1.6)	<0.001
Compensated cirrhosis	58 (4.6)	51 (0.4)	<0.001
Decompensated cirrhosis	34 (2.7)	77 (0.6)	<0.001
Esophageal varices (bleeding)	8 (0.6)	15 (0.1)	<0.001
Ascites	21 (1.7)	69 (0.6)	<0.001
HRS	3 (0.2)	5 (0.0)	<0.001
HE	19 (1.5)	1 (0.0)	<0.001
Chronic liver failure	33 (2.6)	49 (0.4)	<0.001
HCC	16 (1.3)	38 (0.3)	<0.001
Liver transplant	3 (0.2)	3 (0.0)	<0.001
Liver-related death	33 (2.6)	92 (0.7)	<0.001

Abbreviations: HCC = hepatocellular cancer; HE = hepatic encephalopathy; HRS = hepatorenal syndrome, NAFLD = nonalcoholic fatty liver disease.

Table S4. Anatomical Therapeutic Chemical Classification System (ATC) codes for comorbidities.

	**ATC**
Anti-diabetic medication	A10
Anti-hypertensive medication	C03AA, C07AB, C08C, C09
Statins	C10AA

Table S5. C-index for continuous or age-dependent categorical FIB-4 compared to fibrosis stage estimated by liver biopsy or by biopsy or VCTE when biopsy was not available.

**C-index for FIB-4 vs. biopsy (n = 904)**	**C statistics *(95% CI)* at 5 years**	**C statistics *(95% CI)* at 10 years**
**Biopsy**	0.701 *(0.644-0.733)*	0.734 *(0.630-0.785)*
**FIB-4 continuous**	0.713 *(0.589-0.760)*	0.727 *(0.623-0.774)*
**FIB-4 categorical**	0.690 *(0.616-0.722)*	0.716 (*0.626-0.755)*
**C-index for FIB-4 vs. VCTE or Biopsy (n = 1,022)**		
**VCTE or biopsy**	0.724 *(0.634-0.778)*	0.748 *(0.668-0.802)*
**FIB-4 continuous**	0.695 *(0.619-0.736)*	0.703 *(0.612-0.754)*
**FIB-4 categorical**	0.710 *(0.624-0.767)*	0.716 *(0.618-0.764)*

Abbreviations: FIB-4 = fibrosis-4; VCTE = vibration-controlled transient elastography.

FIB-4 category was age-dependent: for age<64: low risk: <1.3, intermediate risk: 1.3-2.67, high risk: >2.67; for age ≥65: low risk: <2.0, intermediate risk: 2.0-2.67, high risk: >2.67
